# Ornaments indicate parasite load only if they are dynamic or parasites are contagious

**DOI:** 10.1093/evlett/qrad017

**Published:** 2023-05-25

**Authors:** Liam R Dougherty, Faith Rovenolt, Alexia Luyet, Jukka Jokela, Jessica F Stephenson

**Affiliations:** Department of Evolution, Ecology and Behaviour, University of Liverpool, Liverpool, United Kingdom; Department of Biological Sciences, University of Pittsburgh, Pittsburgh, PA, United States; Department of Aquatic Ecology, EAWAG, Swiss Federal Institute of Aquatic Science and Technology, Dübendorf, Switzerland; Institute for Integrative Biology, ETH Zürich, Zürich, Switzerland; Department of Aquatic Ecology, EAWAG, Swiss Federal Institute of Aquatic Science and Technology, Dübendorf, Switzerland; Institute for Integrative Biology, ETH Zürich, Zürich, Switzerland; Department of Biological Sciences, University of Pittsburgh, Pittsburgh, PA, United States

**Keywords:** sexual selection, epidemiology, host–parasite interactions, courtship behavior, sexual ornaments, meta-analysis

## Abstract

Choosing to mate with an infected partner has several potential fitness costs, including disease transmission and infection-induced reductions in fecundity and parental care. By instead choosing a mate with no, or few, parasites, animals avoid these costs and may also obtain resistance genes for offspring. Within a population, then, the quality of sexually selected ornaments on which mate choice is based should correlate negatively with the number of parasites with which a host is infected (“parasite load”). However, the hundreds of tests of this prediction yield positive, negative, or no correlation between parasite load and ornament quality. Here, we use phylogenetically controlled meta-analysis of 424 correlations from 142 studies on a wide range of host and parasite taxa to evaluate explanations for this ambiguity. We found that ornament quality is weakly negatively correlated with parasite load overall, but the relationship is more strongly negative among ornaments that can dynamically change in quality, such as behavioral displays and skin pigmentation, and thus can accurately reflect current parasite load. The relationship was also more strongly negative among parasites that can transmit during sex. Thus, the direct benefit of avoiding parasite transmission may be a key driver of parasite-mediated sexual selection. No other moderators, including methodological details and whether males exhibit parental care, explained the substantial heterogeneity in our data set. We hope to stimulate research that more inclusively considers the many and varied ways in which parasites, sexual selection, and epidemiology intersect.

## Teaser

Choosing a mate with few or (ideally) no parasites has many potential benefits. Indeed, good health seems like such an important attribute in a potential mate that many researchers have suggested that exaggerated sexual ornaments have evolved specifically to signal absence of infection, or parasite resistance, in the bearer. A key prediction of these ideas is that, within a population, individuals with the biggest, brightest, most attractive ornaments have the fewest parasites. Here, we summarize decades of tests of this prediction across host and parasite taxa to find overall weak support. In fact, this pattern is only significantly supported when ornaments can change rapidly in response to mate condition or parasites can transmit during mating. We propose that these findings strongly indicate a need to incorporate epidemiology into an updated framework of parasite-mediated sexual selection.

## Introduction

Mate choice can drive the evolutionary trajectory of individual species, the formation of new species, and their loss through hybridization: It has profound implications for animal evolutionary ecology (Rosenthal, 2018). Potential mates vary in quality and choosers benefit from choosing high-quality partners, which intuitively should include mates with no, or few, parasites. However, how parasites affect mate choice remains poorly understood (Balenger & Zuk, 2014). Several non-mutually exclusive hypotheses have been proposed to explain why sexual ornaments might indicate the number of parasites with which a host is infected (“parasite load”; Table 1). By using such ornaments to avoid parasitized mates, choosers may obtain the indirect, genetic benefits of offspring better able to resist parasites (i.e., offspring better able to prevent the establishment or growth of parasite infection [Folstad & Karter, 1992; Hamilton & Zuk, 1982]). Avoiding parasitized mates may also yield the direct benefits of a reduced risk of infection (Able, 1996; Borgia & Collis, 1989, 1990; Loehle, 1997) or a more fecund (Fedorka, 2014), efficient parent (Clayton, 1991; Møller et al., 1999) for their offspring. While there is, therefore, much conceptual support for the processes by which parasites may affect sexual selection, these may only operate in host–parasite systems with particular attributes (Read, 1987; Read & Weary, 1990), and there are important limitations on their empirical detection (Poulin & Vickery, 1993; Read, 1988, 1990).

**Table 1.** Summary of hypotheses of parasite-mediated sexual selection and their assumptions.

Hypothesis, definition, and references	Benefit to chooser	Must these factors be present?			Original formulation predicted negative correlation between ornament quality and parasite load?
		Ornament dynamism	Transmission during sex	Paternal care	
Hamilton–Zuk (Hamilton & Zuk, 1982): coevolutionary dynamics provide negative frequency-dependent selection for resistance genes, and thus genetically determined ability to maintain high-quality ornaments.	Indirect	Yes?^c^	No	No	Yes
Immunocompetence handicap (Folstad & Karter, 1992): the process outlined by Hamilton and Zuk may be mediated by testosterone, which promotes ornament development but has an immunocompetence cost.^a^		No	No	No	Yes
Parental care—resource provisioning/efficient parent (Clayton, 1991; Møller et al., 1999): uninfected mates provide more effective parental care.	Direct	Yes	No	Yes	Yes
Pathogen avoidance (Borgia & Collis, 1989, 1990): ornaments—such as large white patches—aid in the assessment of parasite load.^b^		No	Yes	No	No
Contagion indicator/transmission avoidance (Able, 1996; Loehle, 1997): ornaments honestly signal current parasite load, facilitating contagion avoidance.		Yes	Yes	No	Yes
Fertility assurance (Fedorka, 2014): infection reduces fecundity.		Yes	No	No	Yes

^a^We additionally expect host taxon to be important to this hypothesis, as invertebrates do not produce testosterone.

^b^We expect this hypothesis to only be supported in systems in which the parasite is visible externally, so parasite type (endoparasite vs. ectoparasite) should be important here.

^c^Hamilton and Zuk do not explicitly consider ornament dynamism, but the ornaments they suggest to be important at the intraspecific level are all dynamic to some extent (blood color, plumage “shabbiness,” skin pigmentation, behavioral displays, urine odor). While it is feasible that static ornament quality is affected by parasite infection of the bearer as a juvenile, Hamilton and Zuk suggest that such infections would not be involved in the co-adaptational cycles assumed by their hypothesis.

Nevertheless, primary and meta-analytical research testing the hypothetical role of parasites in sexual selection has focused on the correlation between ornament quality and parasite load within populations, predicted to be negative by most hypotheses (Table 1; Dougherty, 2021a; Garamszegi, 2005; Hamilton & Poulin, 1997; Hernández et al., 2021; Møller et al., 1999; Weaver et al., 2018; White, 2020). Such studies, while often finding a significantly negative overall correlation, highlight the variation in the strength and sign of the relationship (Dougherty, 2021a; Garamszegi, 2005; Hamilton & Poulin, 1997; Hernández et al., 2021; Møller et al., 1999; Weaver et al., 2018; White, 2020). Relevant to the hypotheses in Table 1, Møller et al. (1999) found that correlations were more strongly negative among ectoparasites than endoparasites, but did not observe significant differences between systems with and without paternal care or between behavioral and morphological sexually selected ornaments.

We leverage the several hundred studies published since and apply phylogenetically controlled meta-analytical techniques to a data set almost seven times larger than that of Møller et al. (1999), to evaluate potential moderators of the relationship between parasite load and ornament quality. We control for host, parasite, and study characteristics and focus on ornament dynamism, the risk of parasite transmission during sex, and host paternal care: Together, these moderators discriminate between the prevailing hypotheses of parasite-mediated sexual selection (Table 1). Ornament dynamism ranges widely, from morphological structures that are fixed at sexual maturity, plumage that is renewed between breeding seasons, to courtship behavior that can vary over seconds or minutes. Most hypotheses assume that ornament quality provides choosers with information about a courter’s current or recent parasite infection (Able, 1996; Clayton, 1991; Fedorka, 2014; Hamilton & Zuk, 1982; Loehle, 1997; Møller et al., 1999). For this assumption to be met, ornament quality must be able to change in the short term in response to infection, and many sexually selected ornaments do reflect such changes in condition (Folstad & Karter, 1992; Hill et al., 1999; Stephenson et al., 2020; Wingfield et al., 1990). However, ornaments that are relatively static during sexual maturity may better reveal a courter’s genetic and developmental quality, and may be harder to cheat as they often require longer-term investment (Hill et al., 1999; Stephenson et al., 2020). Additionally, as static ornaments are not affected by exposure to parasites, their ability to reliably signal courter quality is not vulnerable to stochastic variation in that exposure—a key criticism of the concept of parasite-mediated sexual selection (Poulin & Vickery, 1993). Despite these important differences, ornament dynamism is not often considered in studies of parasite-mediated sexual selection (but see Gilbert & Uetz, 2016; Hill et al., 1999; Stephenson et al., 2020), nor is it explicitly addressed in parasite-mediated sexual selection hypotheses. We also test how the correlation between ornament quality and parasite load may be moderated by the risk of the parasite transmitting between partners during sex, and whether hosts have paternal care: Both moderators have long been central to the theory of parasite-mediated sexual selection, and a significant effect of either would indicate the importance of direct benefits to choosers (Table 1).

## Methods

Throughout we follow the recent extension to the PRISMA reporting guidelines for ecology and evolutionary biology (O’Dea et al., 2021). See Supporting Information for a completed PRISMA checklist.

### Literature searches

We searched for published, peer-reviewed papers using three approaches. First, on or before 5 December 2020, we performed informal literature searches using a range of online databases. Second, we obtained all relevant papers included in the reviews by Møller et al. (1999); Garamszegi (2005), and Dougherty (2021a). Third, we searched Web of Science on 9 April 2021, using the following keywords: “TS=(infect* OR parasit*) AND TS=(ornament OR secondary sex* charact* OR sex* display OR court*) NOT TS=(human OR plant),” and added all papers citing Møller et al. (1999) on 10 April 2021. We imported these records into the online tool Rayyan, removed duplicates, and screened the abstracts and titles against our inclusion criteria (Supplementary Figure S1). We then read all relevant articles in full. To be included in the analysis, a study had to (a) present data for sexually mature individuals of a non-human animal species, (b) report within-species variation in a morphological, behavioral, or extended ornament, (c) report some measure of parasite load for the same host individuals, and (d) provide sufficient statistical information for an effect size to be calculated (see Supporting Information).

While parasite-mediated sexual selection hypotheses tend to focus on elaborate male morphological ornaments, such as plumage or bright skin patches, we expand the scope of our data set by (a) considering display behaviors (following Møller et al., 1999) and extended ornaments such as the bowers of bowerbirds because these potentially honestly indicate courter condition or quality (Borgia et al., 2004; Doucet & Montgomerie, 2003a; Dougherty, 2021a) and (b) considering female ornamentation because mating preferences in relation to partner condition and quality are seen in both sexes (Amundsen, 2000; Bonduriansky, 2001; Kraaijeveld et al., 2007; Rosenthal, 2018). Indeed, while male ornaments tend to be more elaborate and conspicuous (Andersson, 1994), a recent meta-analysis of mutually ornamented birds found that ornaments are equally strongly associated with indicators of body condition in both sexes (Nolazco et al., 2022). While most studies quantified ornament quality and parasite load at the same time, we included studies where parasite load and ornament quality were measured at different times (Dawson & Bortolotti, 2006; Hill & Farmer, 2005; Lindström & Lundström, 2000; López, 1998; Stephenson et al., 2020). As with other reviews of this topic, we did not consider sexually selected weapons in our analysis (McCullough et al., 2016).

### Effect sizes

We used Pearson’s correlation (*r*) between parasite load and ornament quality as the effect size (see Supporting Information for extraction methods). We chose the correlation coefficient, instead of the slope of the relationship, as our effect size because it is a better metric of how useful ornament quality may be as an indicator (i.e., a shallow slope with strong correlation means ornament quality is still a reliable indicator of parasite load, but a weak correlation suggests a chooser could easily make a mistake). Here, a positive correlation means that individuals with higher parasite loads have higher quality ornaments, and a negative correlation (such as that predicted by most hypotheses (Table 1)) means that individuals with higher parasite loads have lower quality ornaments. Studies often report nonsignificant results without reporting directional information. Such data are traditionally excluded from meta-analysis, resulting in a bias against the inclusion of nonsignificant results. We found 61 such estimates in our literature search and included them as “directionless” estimates in our analysis by assigning them a correlation of zero (Dougherty, 2021b, 2021a; Harts et al., 2016).

We considered as higher quality ornaments: (a) morphological traits that are larger or more symmetrical; (b) colored plumage or skin patches that are larger, brighter, more saturated, more reflective, or more symmetrical; (c) extended ornaments that are present (e.g., males with or without bowers [Borgia & Collis, 1990]) or larger; and (d) sexual displays that are initiated sooner, last longer, or are more energetic. For roughly half of our effect sizes, we found published evidence that our estimate of quality did correspond to ornament attractiveness to the intended receiver (see “ornament mate choice” in the Moderators section). For the others, we acknowledge that our human estimates of ornament quality may not correspond to ornament attractiveness. With this caveat in mind, we use “quality” to refer to these characteristics of the included ornaments hereafter.

Our analysis included data from 142 studies (Adamo et al., 2014; Aguilar et al., 2007; An & Waldman, 2016; Baeta et al., 2008; Barber, 2002; Biard et al., 2010; Blanco et al., 1999; Borgia & Collis, 1989; Borgia et al., 2004; Bortolotti et al., 2009; Bosholn et al., 2016; Brønseth & Folstad, 1997; Buchanan et al., 1999; Buchholz, 1995; Candolin & Voigt, 2001; Chappell et al., 1997; Chemnitz et al., 2015; Clayton, 2015; Cook et al., 2013; Córdoba-Aguilar, 2002; Córdoba-Aguilar et al., 2003; Costa & Macedo, 2005; Dale et al., 1996; Darolovà et al., 1997; De Lisle & Rowe, 2015; Doucet & Montgomerie, 2003b, 2003c; Dufour & Weatherhead, 1998; Dufva & Allander, 1995; Edler & Friedl, 2010; Edler et al., 2004; Engen & Folstad, 1999; Fenoglio et al., 2004; Figuerola et al., 2003; Fitzgerald et al., 1994; Folstad et al., 1994, 1996; Garamszegi, 2005; Garamszegi et al., 2005; Gibson, 2015; Gilbert & Uetz, 2016; Gilbert et al., 2016; Greenspan et al., 2016; Gunderson et al., 2009; Harper, 1999; Hatchwell et al., 2001; Hausfater et al., 2015; Henschen et al., 2017; Hill & Brawner, 1998; Höglund et al., 1992; Hõrak et al., 2001, 2004; Houde & Torio, 1992; Hund et al., 2021; Kekäläinen et al., 2011, 2014; Kennedy et al., 1987; Kopena et al., 2020; Korpimäki et al., 1995; Kortet & Taskinen, 2004; Kose & Møller, 1999; Kose et al., 1999; Lagesen & Folstad, 1998; Lai et al., 2016; Lindström & Lundström, 2000; Llanos-Garrido et al., 2017; López, 1998; Madelaire et al., 2013; Magalhães et al., 2014; Markusson & Folstad, 1997; Martín et al., 2008; Martínez-Padilla et al., 2007; McGraw & Hill, 2000; McLennan & Shires, 1995; Megía-Palma et al., 2016; Merilä et al., 1999; Merrill et al., 2015; Milinski & Bakker, 1990; Møller, 1990, 1991a, 1991b, 2000, 2002; Møller et al., 1999; Molnár et al., 2012; Moreno-Rueda, 2005; Mougeot et al., 2005, 2007, 2009a, 2009b, 2010, 2016; Müller & Ward, 2010; Mulvey & Aho, 1993; Nordeide et al., 2008; Ottová et al., 2005; Pélabon et al., 2005; Pérez-Tris et al., 2002; Petrie et al., 1996; Pfennig & Tinsley, 2002; Piersma et al., 2001; Potti & Merino, 1996; Pröhl et al., 2013; Pruett-Jones et al., 2015; Redpath et al., 2000; Rodrigo et al., 2016; Roulin et al., 2001; Saino & Møller, 1994, 1996; Saino et al., 1995; Schall, 1986; Schall & Dearing, 1987; Setchell et al., 2006; Seutin, 1994; Shawkey et al., 2007, 2009; Simmons, 1994; Siva-Jothy, 2000; Skarstein & Folstad, 1996; Skarstein et al., 2005; Stephenson et al., 2020; Sundberg, 1995; Surmacki et al., 2015; Taggart & Schultz, 2017; Taylor et al., 1998; Thompson et al., 1997; Trigo & Mota, 2016; Tripet & Richner, 1999; Václav et al., 2007; Vergara et al., 2012; Votýpka et al., 2003; Warner & Schultz, 1992; Weatherhead et al., 1993; Wedekind, 1992; Weiss, 2006; Wiehn et al., 2010; Yang et al., 2013; Zirpoli et al., 2013; Zuk, 1987; Zuk et al., 1998).

### Moderators

We collected data on 12 categorical variables that we expected might moderate the correlation between parasite load and ornament quality:

1. *Host taxon*. We categorized hosts into seven taxonomic groupings: arachnids, insects, fish, amphibians, reptiles, birds, and mammals. A stronger relationship between ornament quality and parasite load among vertebrates would support the immunocompetence handicap hypothesis (Folstad & Karter, 1992).
2. *Host sex*. We predicted that the relationship between ornament quality and parasite load would be stronger for males because males tend to have more elaborate ornaments than females (Andersson, 1994).
3. *Host paternal care* (males only). For each host species, we determined whether males contributed to parental care (biparental or paternal care species) or not (maternal or no care species). We predicted that the relationship between male ornament quality and parasite load would be strongest for species with paternal care, as in these species females may face both direct and indirect costs from choosing highly parasitized mates.
4. *Parasite taxon*. We categorized parasites into one of 10 taxonomic groups: viruses, bacteria, fungi, protists, nematodes, platyhelminthes, acanthocephalans, cnidarians, bivalve mollusks, and arthropods (including mites, ticks, lice, and parasitic flies). We had no clear prediction for this moderator.
5. *Parasite type* (morphological traits only). We considered parasites found inside the host body or cells (endoparasites, including viruses that infect blood cells) and parasites that live on the exterior of the host body (ectoparasites). We predicted that ectoparasites would be more strongly related to morphological ornament quality as they have the potential to directly degrade morphological ornaments (especially plumage; Table 1).
6. *Parasite sexual transmission risk*. We classified parasites according to the extent to which transmission between hosts during sexual interactions is likely. We considered sexual transmission to be a low risk for parasites that are exclusively transmitted by vectors, parasites that shed noninfective life stages, parasites that only parasitize juvenile hosts, and parasites that have obligately multihost lifecycles (though we acknowledge that in territorial breeders, choosers of mates infected with such parasites may be at increased risk of infection [Hund et al., 2021; Zelmer et al., 1999]). We considered sexual transmission to be a medium risk for parasites that transmit via the water column, host feces, or shared space. We considered sexual transmission to be a high risk for parasites that transmit via host–host physical contact, those that actively transmit between hosts in proximity, and blood parasites that transmit during sex. We predicted that the relationship between ornament quality and parasite load would be strongest for parasites with a high risk of sexual transmission, as here choosers may benefit both directly and indirectly from avoiding parasitized partners.
7. *Ornament evidence for mate choice*. We considered an ornament to be implicated in mate choice if the study authors supported this assertion with empirical data within the study in question, or from another peer-reviewed publication. We predicted that ornaments that have a demonstrated role in mate choice should show a stronger relationship with parasite load than those without but acknowledge that absence of evidence is not evidence of absence.
8. *Ornament dynamism score*. We scored the extent of ornament dynamism using a 3-point scale (Table 2). We predicted that the relationship between current parasite load and ornament quality should be strongest for the most dynamic ornaments.
9. *Ornament dynamism*. Because the number of data points we obtained for the 0 category of the dynamism score was relatively small, we created a binary variable by considering traits in category 2 as dynamic, and those in categories 0 and 1 as not dynamic (Table 2). We predicted that the relationship between current parasite load and ornament quality should be stronger for dynamic ornaments.
10. *Parasite measurement*. We compared studies that used continuous or categorical measure of parasite counts (intensity), compared hosts with or without parasites (presence/absence), or compared hosts between different experimental treatments (experimental group). We had no clear prediction for this moderator.
11. *Uninfected hosts present*. We recorded whether the correlation was calculated including individuals that were uninfected. We predicted that the relationship between ornament quality and parasite load would be strongest for studies that included some uninfected hosts in the analysis, as the absolute range in parasite load and ornament quality may be larger in these samples.
12. *Study type*. We considered three types of study: observational studies that quantify the parasite load of wild-caught animals, experimental studies that manipulate host–parasite load by adding or removing parasites, and experimental studies in which individuals are experimentally infected and then parasite load is measured at some point in the future (“resistance” studies). We predicted that the relationship between ornament quality and parasite load would be strongest for studies that experimentally manipulate parasite load because these control for variation in exposure.

**Table 2.** Classification scheme for ornament dynamism.

Dynamism score	Examples of ornaments	Binary category
0: Develop once and are fixed throughout sexual maturity	• Fixed morphological traits (e.g., comb size in fowl) • Feather and skin coloration that is stable at maturity	Static
1: Change gradually after sexual maturity, but little within a breeding season	• Morphological traits that change between breeding seasons (e.g., reindeer antlers) • Feather color area • Size of plumage	
2: Typically vary within a breeding season, including on the scale of minutes	• Morphological traits which change within a breeding season (e.g., primate sexual swellings) • Feather coloration/UV reflectance (often relies on preening, and may be directly impacted by parasites) • Skin pigmentation • Pheromone composition • Behavior • Extended traits (e.g., nests and bowers of birds)	Dynamic

### Statistical analysis

We used three data sets to address our questions and assess the robustness of our results. The first included all correlations (full data set, *k* = 424). We used the second, which included only correlations with directional information (*k* = 363), to test the sensitivity of our results to the inclusion of directionless estimates. We used the third, which included only correlations considering male morphological traits, and where parasite load and ornaments were measured at the same time (*k* = 259), to test the sensitivity of the results to our broad inclusion criteria (see Supplementary Figure S2).

In order to account for nonindependence of correlations from closely related host species, we constructed a supertree for the host species in the data set using the Open Tree of Life database (Hinchliff et al., 2015), combining available phylogenetic and taxonomic information (Hadfield & Nakagawa, 2010). Trees were created using the Rotl v3.0.12 (Michonneau et al., 2016) and Ape v5.6 (Paradis et al., 2004) R packages. Given the absence of accurate branch length data, branch lengths were first set to one and then made ultrametric using Grafen’s method (Grafen, 1989). When analyzing a subset of the data, we used an appropriately pruned tree. Supplementary Figure S3 shows the ultrametric tree for the full data set.

All statistical analyses were performed using R v.4.1.2 (R Core Team, 2020) and the package Metafor v.3.4 (Viechtbauer, 2010). To determine the overall correlation between ornament quality and parasite load, we ran a multilevel random-effects model with host phylogeny, host species, study, and an observation-level identifier (“observation ID”) as random factors, using the rma.mv function in Metafor. Host phylogeny was incorporated into the model using a correlation matrix, assuming that traits evolve via Brownian motion. Study was included as a random factor because we obtained more than one correlation from most studies (mean = 2.9, range = 2–21). We ran this model separately for the three data sets. We ran an additional model using the full data set, in which we added a random factor (“experiment ID”) to account for the potential nonindependence of correlations measured using the same set of host individuals (Noble et al., 2017). We converted this factor into a within-study covariance matrix, assuming estimates using the same host individuals have a correlation of 0.5.

We used meta-regression models to examine the effect of our moderator variables on the average correlation (Nakagawa & Santos, 2012), using the rma.mv function in Metafor. Each model included host phylogeny, host species, study, and observation ID as random factors as before, but now also included as fixed factors one of the 12 categorical moderator variables. We tested post hoc for an interaction between ornament dynamism and sexual transmission risk using a model with these two factors, and their interaction, included as fixed factors. To test whether the average correlation significantly differed between moderator categories, we used the *Q*_*M*_ statistic, with a significant value indicating that the moderator accounts for a significant proportion of the between-study heterogeneity (Koricheva et al., 2013). We calculated the amount of variance explained by the fixed effect (marginal *R*^2^) for each model using the orchaRd R package (Nakagawa et al., 2021). We additionally ran these models with the intercept term dropped to obtain estimates of the average correlation for each categorical moderator level (in effect running a separate meta-analysis for each moderator level: Supplementary Table S1). To improve our ability to detect biologically relevant differences, we excluded any trait categories with ten or fewer data points when performing meta-regressions. Finally, we tested for two types of publication bias in the data set (see Supporting Information).

## Results

**Figure 1.**
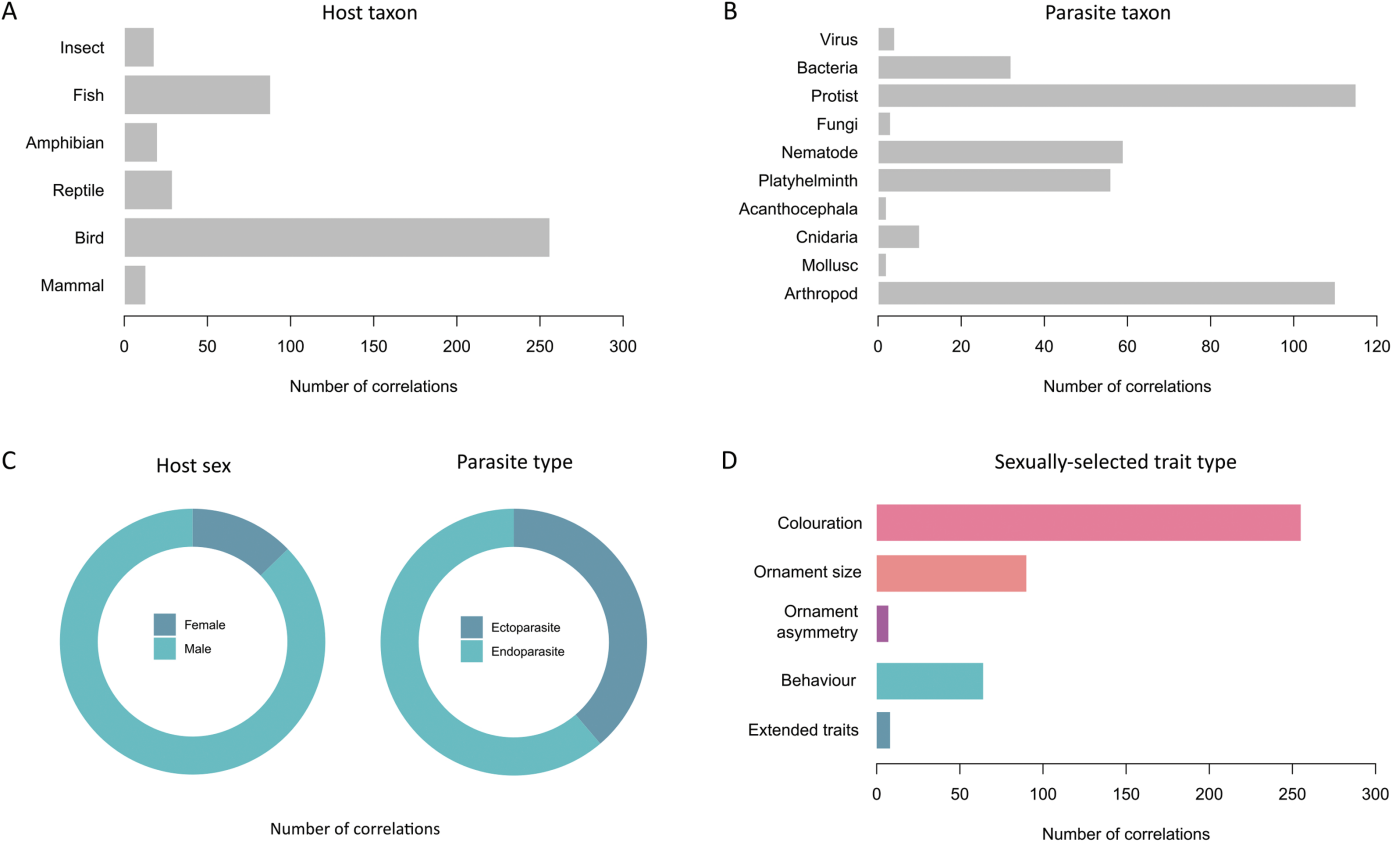
Summary of the host (A) and parasite (B) taxa, host sex and parasite type (C), and the type of ornament (D) represented in our data set. Histograms (A, B, D) and pie charts (C) of the number of correlations in our data set corresponding to each host (A) and parasite (B) taxon, each host sex and parasite type (C), and type of ornament (D).

**Figure 2.**
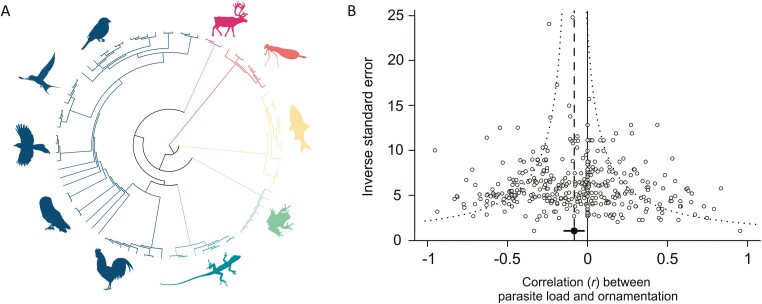
Across taxa, there is a significantly negative correlation between parasite load and ornament quality. (A) Phylogeny of hosts in our data set: insects and arachnids, fish, amphibians, reptiles, birds, and mammals. (B) Funnel plot of the correlation between ornament quality and parasite load plotted against the inverse standard error (larger values represent studies with larger sample sizes) for the full data set (*k* = 424). The filled point and dashed vertical line show the meta-analytic mean, with corresponding 95% confidence interval (error bars) and 95% pseudo-confidence region (dotted lines).

We tested whether ornament quality reliably indicates parasite load by assembling 424 correlations from 142 studies, 83 host species, and 10,663 host individuals (Figures 1 and 2A). Overall, individual hosts with higher parasite loads had lower quality ornaments (mean *r* = −.084, 95% CI = −0.143 to −0.023, *k* = 424; Figure 2B, Supplementary Table S3). The overall result remained unchanged after removing directionless estimates (mean *r* = −.083, 95% CI = −0.152 to −0.013, *k* = 363) and after incorporating a covariance matrix to account for the potential for estimates from the same host individuals to be correlated (mean *r* = −.089, 95% CI = −0.148 to −0.030, *k* = 424), but not after simultaneously removing correlations from females, non-morphological traits, and cases where parasites and ornaments were measured at different times (mean *r* = −.07, 95% CI = −0.143 to 0.004, *k* = 259). Because this subset represents a substantial reduction in sample size, we evaluated whether the loss of statistical power explains the difference in results by randomly removing 165 effect sizes and re-running the analysis. Among 1,000 such re-analyses, the *p* value was greater than .05 in 35.8% of cases (Supplementary Figure S4), and the overall mean correlation between parasite load and ornament quality was −0.083 (bootstrapped 95% CI: −0.119 to −0.017). The full data set was characterized by high heterogeneity (total *I*^2^ = 82.5%). Partitioning of this heterogeneity indicated that a negligible amount of variation was attributable to phylogenetic relatedness (0%), while 21.4%, 25.6%, and 35.5% were attributable to species-level, study-level, and observation-level differences, respectively.

**Figure 3.**
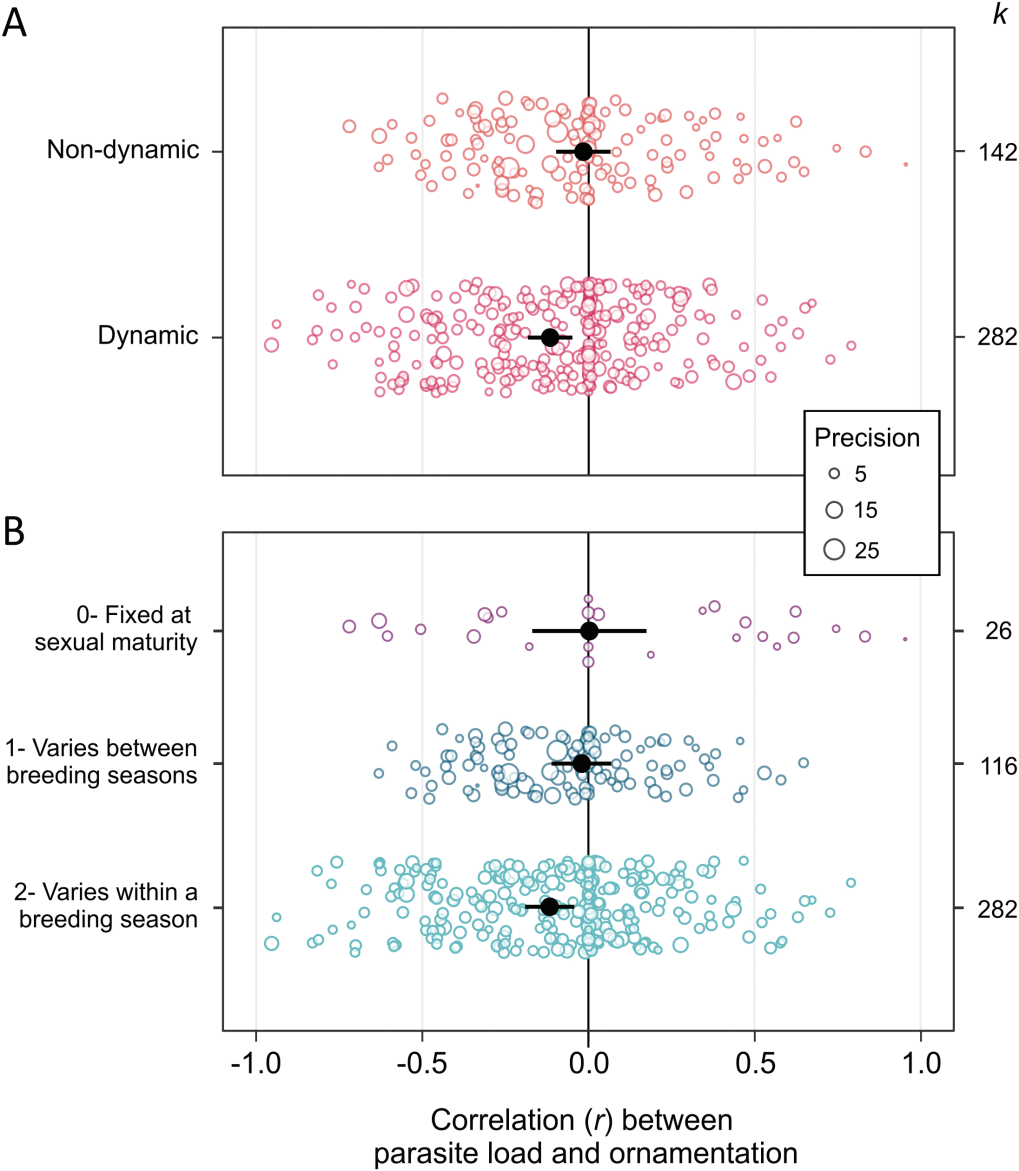
The quality of dynamic ornaments is more negatively correlated with parasite load than that of non-dynamic, or static, ornaments. We considered dynamism on a binary scale (A) and on a 3-point scale (B; see Table 2). Open points are scaled according to study variance (precision). Filled points represent the meta-analytic means for each category, and bars show the 95% confidence interval. *k* = number of effect sizes for each category.

**Figure 4.**
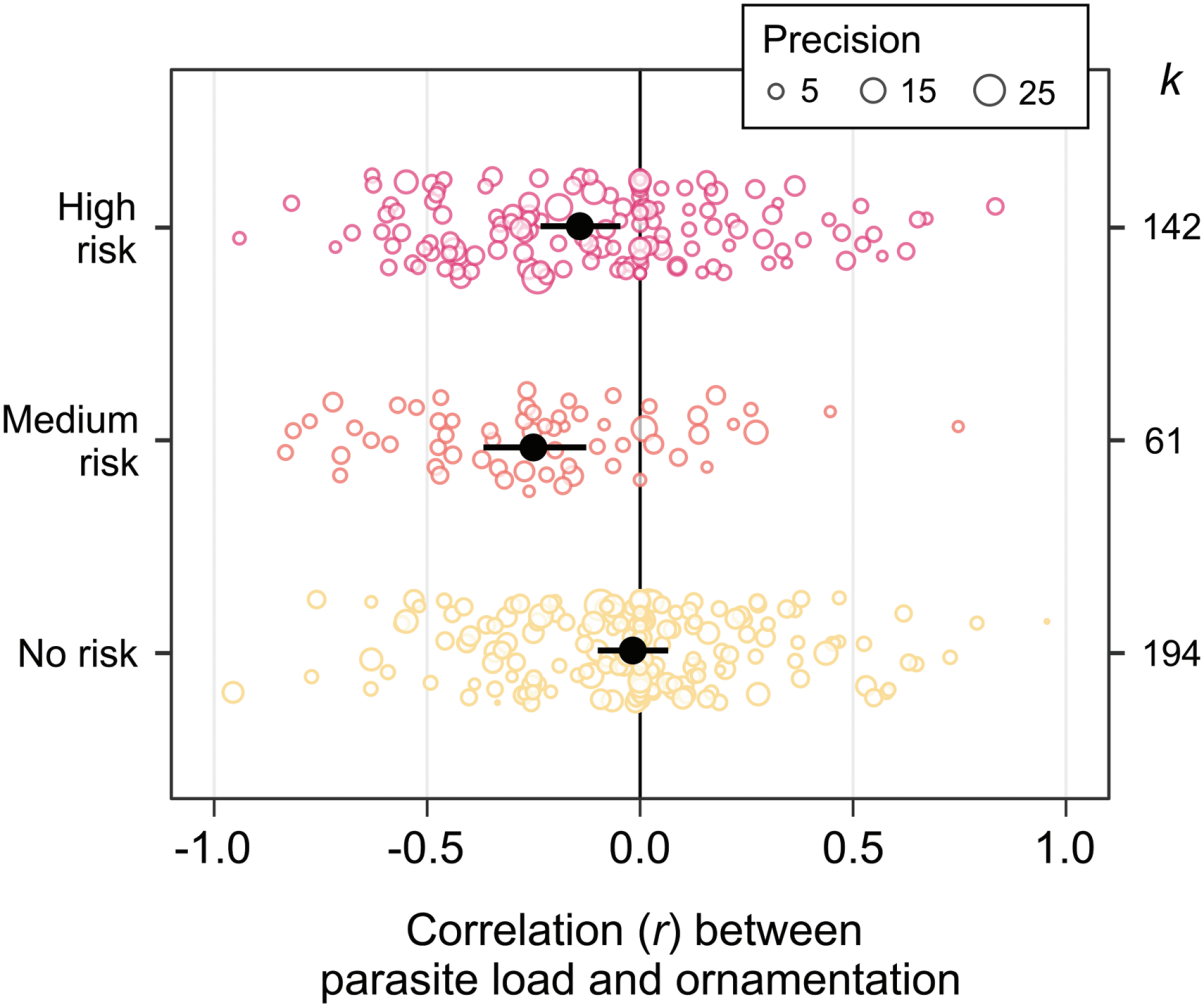
Ornament quality and parasite load were more negatively correlated among parasites that could transmit during sex. Open points are scaled according to study variance (precision). Filled points represent the meta-analytic means for each category, and bars show the 95% confidence interval. *k* = number of effect sizes for each category.

Next, we tested whether variation in the correlation between parasite load and host ornament quality could be explained by the moderator variables highlighted in Table 1. The relationship between parasite load and host ornament quality depended on ornament dynamism when considered as a binary factor (*Q*_*M* 1_ = 5.37, *p* = .02, marginal *r*^2^ = 0.021, *k* = 424), but not as a 3-point dynamism score (Dynamism score; *Q*_*M* 2_ = 5.42, *p* = .07, marginal *r*^2^ = 0.021, *k* = 424): Dynamic ornament quality was significantly associated with lower parasite load, whereas static ornament quality was not (Figure 3). The relationship between parasite load and host ornament quality depended on the risk of sexual transmission of parasites (*Q*_*M* 2_ = 10.87, *p* = .004, marginal *r*^2^ = 0.07, *k* = 397): Parasites with a medium or high risk of sexual transmission were significantly associated with lower quality ornaments, whereas parasites with no risk of sexual transmission were not (Figure 4). There was no significant interaction between ornament dynamism (as a binary trait) and the risk of sexual transmission (*Q*_*M* 2_ = 0.32, *p* = 0.85, *k* = 397). The relationship between parasite load and ornament quality was not significantly moderated by a range of ecological, biological, or methodological variables (Supplementary Tables S1 and S2). We also found little evidence for publication bias: The relationship between parasite load and host ornamentation was not related to study publication year (β = 0.003, 95% CI = −0.003 to 0.009) or sample size (β = 0.002, 95% CI = −0.011 to 0.017; Supplementary Table S1).

## Discussion

Overall, we found a weak but significantly negative correlation between individual parasite load and ornament quality across 424 effect sizes, with no signs of publication bias (Figure 2). Our effect size is similar to that found by the second largest meta-analysis on this topic (Møller et al., 1999; *k* = 62, *r* = −.123, 95% CI = −0.152 to −0.095), while meta-analyses using 8–30 estimates have found larger effect sizes (Garamszegi, 2005; Hamilton & Poulin, 1997; Weaver et al., 2018). There was a large amount of heterogeneity in the data set, significant portions of which were explained by two of our focal moderators. We found that the quality of dynamic ornaments was negatively correlated with parasite load, whereas the overall correlation among static ornaments was not significantly different from 0 (Figure 3). Additionally, the correlation between ornament quality and parasite load was only significantly negative among parasites that could transmit during sex (Figure 4). We found no evidence that male engagement in parental care, host taxon, or whether parasites were ectoparasitic or endoparasitic explained variation in this relationship. Together, these results suggest that parasites mediate sexual selection through the direct benefits to the chooser and help to discriminate between the hypotheses listed in Table 1. Importantly, while these results represent the most comprehensive evaluation to date of a key prediction of many parasite-mediated sexual selection hypotheses, we must acknowledge an important caveat. We follow the authors of most included studies and use “ornament quality,” estimated by human observers, as a proxy for “ornament attractiveness” to the target audience of choosers. Whether the ornament had been demonstrated as important in mate choice did not moderate our result, but our results may still be an unreliable estimate of how actual mate attractiveness correlates with parasite load. With this caveat in mind, we discuss our results and their implications for our understanding of the role of parasites in sexual selection. We suggest explanations for, and potential future research directions to tackle, the remaining heterogeneity.

We found that the overall correlation between parasite load and ornament quality was significantly, but weakly, negative. Several non-mutually exclusive processes may obscure the hypothesized correlation between ornament quality and parasite load and contribute to the heterogeneity in our data set. First, perhaps focal parasites are not virulent enough to warrant discrimination against infected mates (Hawley et al., 2021; Hund et al., 2021; Knell, 1999; Read, 1988), or the host’s maintenance of high-quality ornaments despite infection represents successful manipulation by the parasite to maintain transmission opportunities (Abbot & Dill, 2001; Adamo et al., 2014; Burand et al., 2005; Heil, 2016). Virulence is incredibly challenging to quantify in natural communities (Walsman et al., 2022); these data are available for few if any of the parasites in our data set. Second, because parasites are typically aggregated among hosts in a population (Shaw et al., 1998), when courters are uninfected, choosers cannot know if this is because they are genetically resistant or not yet exposed (Endler & Lyles, 1989; Poulin & Vickery, 1993; Read, 1990). However, if such variation in exposure obscures the correlation between parasite load and ornament quality, we would anticipate stronger negative correlations among experimental (i.e., those that manipulate exposure) than observational studies, as in Møller et al.(1999), and the inclusion of uninfected individuals in observational studies to moderate the correlation. We found no support for either prediction. Finally, a lack of correlation may result from a decoupling of variation in genetic resistance from variation in parasite load or variation in ornament quality from variation in parasite load. For example, acute infections may change so rapidly that resistance, ornament quality, and load are decoupled (Fedorka, 2014; Read, 1988). That the correlation was only significantly negative among dynamic ornaments, that is, those that can change rapidly in response to short-term changes in courter condition, perhaps supports this idea. Furthermore, the significance of the overall correlation was particularly sensitive to the removal of asynchronous parasite load and ornament quality measurements (Supplementary Table S3), suggesting the timing of parasite infection and ornament development may be a key determinant of ornament reliability as a signal of parasite infection or resistance.

Other processes could drive a positive relationship between parasite load and ornament quality, and approximately 35% of the effect sizes we extracted from the literature were positive (147 of 424; vs. 51% negative). First, courters may “terminally invest”: individuals at a high risk of death may benefit from maximizing their short-term reproductive success at the expense of survival (Clutton-Brock, 1984; Duffield et al., 2017). However, a recent meta-analysis found little evidence for terminal investment in sexual signaling behavior across animals (Dougherty, 2021a). Second, sexually transmitted parasites could manipulate their hosts to invest more into ornaments to increase their own transmission, but this idea has again received little support (Dougherty, 2021a; Poulin, 2010). Finally, in some systems, hosts may choose mates based not on parasite resistance but tolerance (Pfennig & Tinsley, 2002), which is the ability to minimize the per-parasite fitness cost of infection, rather than limiting parasite numbers (Råberg et al., 2007). This idea has received little explicit attention, perhaps due to the challenge of collecting host fitness and parasite load data in natural populations, but several authors have noted that higher quality mates may have more parasites (Endler & Lyles, 1989; Foo et al., 2017; Getty, 2002). Such sexual selection for tolerance could overwhelm the negative frequency-dependent selection on resistance proposed by Hamilton and Zuk (1982), as tolerance is expected to spread in a positive-feedback manner (Roy & Kirchner, 2000).

We found that ornament dynamism was an important moderator of the correlation between ornament quality and parasite load, in support of several hypotheses for the role of parasites in sexual selection (Table 1). However, static ornaments reliably indicated infection load in the two experimental studies that explicitly compared the correlation between parasite load and the quality of both static and dynamic ornaments (Gilbert & Uetz, 2016; Stephenson et al., 2020). One explanation for the lack of significantly negative correlation with static ornaments among our mostly observational effect sizes could be epidemiological: If parasites can transmit through sexual contact but the ornament is static, the sexiest mates may become the most infected by virtue of their high contact rate (Hawley et al., 2011; Knell, 1999), but their ornaments would by definition not reflect their high parasite load. If they maintain high contact rates, such mates may become “sexy superspreaders.” Our ornament dynamism result again underscores the overlooked importance of timing, both of parasite infection and the courter’s investment in ornament quality, to signal reliability.

The risk of parasite transmission during sex was the strongest moderator we identified, which supports hypotheses invoking the direct benefit of avoiding infection (Able, 1996; Borgia & Collis, 1989, 1990; Loehle, 1997). However, we did not find a significant interaction between dynamism and sexual transmission, suggesting that dynamic ornaments can also indicate a host’s load of parasites that cannot transmit during sex. While our analysis may lack the power to detect such an interaction, these best available data therefore suggest that parasites that cannot transmit during sex still impact sexual selection, thus perhaps highlighting the importance of indirect benefits to choosers (Read, 1988). The relative importance of direct and indirect benefits of mating decisions to the process of sexual selection has received substantial theoretical (Fry, 2022) and empirical (Achorn & Rosenthal, 2020; Kelly & Adam-Granger, 2020; Madjidian et al., 2020) testing, with overall mixed results. Intriguingly, and in accordance with our results, the importance of the indirect genetic benefit of offspring parasite resistance appears to be consistently supported as a driver of sexual selection (Achorn & Rosenthal, 2020; Cally et al., 2019; Joye & Kawecki, 2019; Prokop et al., 2012).

Our finding that only parasites that can transmit during sex appear to mediate sexual selection may seem counter to previous findings: Many suggest that sexually transmitted parasites are least likely to mediate sexual selection, since host and parasite interests align to conceal the infection to maximize mating or transmission success (Ewald, 1995; Heil, 2016; Knell, 1999). The resulting selection against virulence should also reduce selection for discrimination against infected mates (Fedorka, 2014; Hawley et al., 2021; Knell, 1999; Read, 1988). The apparent discrepancy between our results and these well-established ideas may be because we included all parasites that could *potentially* transmit during sex (Collier et al., 2022). Most of these primarily transmit through space sharing or nonsexual contact between hosts, which could serve to relax selection against virulence and thus maintain the correlation between the load of these parasites and their host’s ornament quality. Indeed, we found that parasites we classified as posing a medium risk of transmission during sex tended to have a more negative correlation with ornament quality than those posing a high risk.

In conclusion, our result that ornament dynamism and the risk of transmission during sex significantly modify the correlation between ornament quality and parasite load suggests that the direct benefits of avoiding parasitized mates predominantly underlie the role of parasites in sexual selection. However, there is much left to uncover. For example, data on the heritability of parasite resistance are becoming more available and may reveal conditions under which indirect benefits are more important (Balenger & Zuk, 2014). Additionally, our results indicate that the timing, relative to ornament development, and dynamism of infection may be a crucial, overlooked, direction for future research. Overall, we suggest that our ability to explain the heterogeneity in the sign and strength of the correlation between ornament quality and parasite load, exemplified by our data set, has been hampered by the overwhelming research focus on a few of the hypotheses in Table 1. We hope to stimulate research that more inclusively considers the many and varied ways in which parasites, sexual selection, and epidemiology intersect.

## Supplementary Material

qrad017_suppl_Supplementary_Material

## Data Availability

Our data and code are available in the supplement and on Dryad, https://doi.org/10.5061/dryad.kkwh70s8g
