# Supplementary material for: Ornaments indicate parasite load only if they are dynamic or parasites are contagious
Source: Evol Lett. 2023 May 25;7(3):176–90. doi: 10.1093/evlett/qrad017 (PMC10210455; doi:10.1093/evlett/qrad017)
Supplement: qrad017_suppl_Supplementary_Material [file qrad017_suppl_supplementary_material.pdf]

Supporting Information for "Ornaments indicate parasite load only if they are dynamic, or parasites are contagious"

Liam R. Dougherty[1], Faith Rovenolt[2], Alexia Luyet[3,4], Jukka Jokela[3,4], Jessica F. Stephenson[2,5]

[1]*Department of Evolution, Ecology and Behaviour, University of Liverpool, Liverpool, UK*

[2]*Department of Biological Sciences, University of Pittsburgh, Pittsburgh, PA 15260 USA*

[3]*Department of Aquatic Ecology, EAWAG, Swiss Federal Institute of Aquatic Science and Technology, 8600 Dübendorf, Switzerland*

[4]*Institute for Integrative Biology, ETH Zürich, 8092 Zürich, Switzerland*

[5]*Author for correspondence: jess.stephenson@pitt.edu*

Methods

Criteria for study inclusion

We did not consider studies comparing ornaments and parasite load at the interspecific level. We considered ornaments of both sexes, regardless of mating system or sex roles. We relied on the study authors to identify traits that function as sexual ornaments and thus did not limit ourselves to examples where such a function has been confirmed experimentally. We did not consider weapons or other traits that function primarily during intrasexual competition, but we did include traits that were suggested to function in both inter- and intrasexual selection (e.g. badges of status, antlers in reindeer), which is probably very common (Berglund *et al.*, 1996). We excluded studies in which no male ornament was directly measured.

We considered five main categories of ornament: 1) the size of morphological ornaments, including plumage, skin growths, sexual swellings, and antlers; 2) the asymmetry of morphological ornaments; 3) the colouration (including brightness, intensity, hue, saturation,

tone, and reflectance) of plumage or skin patches that have a sexual signalling function; 4) extended ornaments such as nests or bowers; 5) sexual display behaviours. We considered both long-range display behaviours which may be expressed in the absence of members of the opposite sex (such as calling in frogs and crickets) as well as short-range courtship behaviours targeted at a specific individual. Following Dougherty (2021a, 2021b), we focused on behavioural traits that represent either the energy or time invested into signal production (e.g. display duration, rate or intensity) or the motivation to signal (e.g. display latency). We excluded measures of signal complexity (e.g. song repertoire size) or signal composition that do not clearly relate to differences in energetic investment.

We considered all parasite taxa, including viruses, bacteria, single-celled protists and fungi, nematodes, platyhelminths, arthropods, and other animals. As metrics of parasite load, we accepted any quantification of parasite number measured at the individual host level. Because of the typically substantial variation between individual hosts in the impact of infection, even with the same number of parasites, we excluded cases where a disease symptom was measured (e.g. virus-induced lesions (Kortet *et al.*, 2003)) rather than actual parasite load, unless there was a convincing correlation between symptoms and number of parasite individuals. We included experimental studies (i.e., those that use experimental exposure, or removal of parasites from naturally infected hosts) and observational studies (i.e., those that examined natural variation in parasite prevalence). The majority of included studies measured sexual ornaments and parasite load at the same time. However, we also included studies where parasite load and ornament quality were measured at different times (López, 1998; Lindström & Lundström, 2000; Hill & Farmer, 2005; Dawson & Bortolotti, 2006; Stephenson *et al.*, 2020), and studies in which hosts were assigned to different experimental infection groups, but individual parasite load not subsequently measured (e.g. Milinski & Bakker, 1990; Simmons, 1994; Zuk et al., 1998; Gilbert et al., 2016).

Calculating effect sizes

We obtained correlations directly from the meta-analyses by Møller et al. (1999), Garamszegi (2005), and Dougherty (2021a), or from the original papers. Where correlations were not available in the text, we either: 1) converted the results of statistical tests into the correlation coefficient using the formulae in Koricheva et al. (2013) (pp 200-201); 2) calculated them from raw data made available by the authors or extracted from figures using the online tool WebPlotDigitizer v4 (https://apps.automeris.io/wpd/); or 3) calculated them from summary statistics presented in the text or extracted from figures. When sexual trait expression was presented as continuous data, but parasite load was presented as categorical data, we first calculated the standardised mean difference (Hedges' *d*) between two groups. If parasite data consisted of more than two groups, we compared sexual trait expression between the highest and lowest parasite load categories. We then converted the standardized difference between two groups into the correlation coefficient using the equation in Koricheva et al. (2013) (pp 201). When estimates came from a paired experimental design (for example using a test statistic from a paired t-test), we assumed that measurements from the same individual had a correlation of 0.5. In this case, the effect size calculations are identical to those derived from independent-measures tests (Koricheva *et al.*, 2013). We extracted all relevant effect sizes from each paper. This often resulted in multiple correlations per paper, either because studies reported data relating to multiple experiments, populations, or host species, or presented correlation between multiple ornaments or parasite species.

Testing for publication bias

We tested for publication bias in two ways. First, we tested for a temporal change in the average correlation using a meta-regression with study year as a continuous fixed effect (and the same four random effects as above). A significant change in effect size over time has been suggested to represent publication bias (Jennions & Møller, 2002; Koricheva *et al.*, 2013), although other changes in research practices may also play a role. Second, we

tested for funnel plot asymmetry, which can be caused by publication bias against studies with small sample sizes or non-significant results (Koricheva *et al.*, 2013; Nakagawa *et al.*, 2021), using a meta-regression with study precision (inverse standard error) as a fixed effect (Moran *et al.*, 2021; Nakagawa *et al.*, 2021), and the same four random effects as above.

Statistical Analysis

Prior to analysis, all correlations were converted into the Fisher's Z transformation of the correlation coefficient (*Zr*) (Koricheva *et al.*, 2013). We used *Zr* as the response variable for all analyses, and converted results back to *r* for presentation. The associated variance for *Zr* was calculated as 1/(*n* – 3) (Borenstein *et al.*, 2021), with *n* being the total number of animals used in the test. The mean correlation was considered significantly different from zero if the 95% confidence intervals did not overlap zero. We calculated heterogeneity across each dataset using the I^2 statistic (Higgins *et al.*, 2003). We also partitioned heterogeneity with respect to each of the four random factors, following Nakagawa & Santos (2012). I^2 values of 25, 50 and 75% are considered low, medium and high respectively (Higgins *et al.*, 2003).

References

Berglund, A., A. Bisazza & A. Pilastro. (1996) Armaments and ornaments: an evolutionary explanation of traits of dual utility. *Biol. J. Linn. Soc. Lond.*, **58**, 385–399.
Borenstein, M., L.V. Hedges, J.P.T. Higgins & H.R. Rothstein. (2021) *Introduction to meta-analysis*. John Wiley & Sons.
Dawson, R.D. & G.R. Bortolotti. (2006) Carotenoid-dependent coloration of male American kestrels predicts ability to reduce parasitic infections. *Naturwissenschaften*, **93**, 597–602.
Dougherty, L.R. (2021a) Meta-analysis reveals that animal sexual signalling behaviour is honest and resource based. *Nat Ecol Evol*, **5**, 688–699.
Dougherty, L.R. (2021b) Meta-analysis shows the evidence for context-dependent mating behaviour is inconsistent or weak across animals. *Ecol. Lett.*
Garamszegi, L.Z. (2005) Bird song and parasites. *Behav. Ecol. Sociobiol.*, **59**, 167–180.
Higgins, J.P.T., S.G. Thompson, J.J. Deeks & D.G. Altman. (2003) Measuring inconsistency in meta-analyses. *BMJ*, **327**, 557–560.
Hill, G.E. & K.L. Farmer. (2005) Carotenoid-based plumage coloration predicts resistance to a novel parasite in the house finch. *Naturwissenschaften*, **92**, 30–34.
Jennions, M.D. & A.P. Møller. (2002) Publication bias in ecology and evolution: an empirical assessment using the 'trim and fill' method. *Biol. Rev. Camb. Philos. Soc.*, **77**, 211–222.
Koricheva, J., J. Gurevitch & K. Mengersen. (2013) *Handbook of meta-analysis in ecology and evolution*. Princeton University Press.

Kortet, R., A. Vainikka & J. Taskinen. (2003) Effect of epidermal papillomatosis on survival of the freshwater fish *Rutilus rutilus*. *Dis. Aquat. Organ.*, **57**, 163–165.
Lindström, K. & J. Lundström. (2000) Male greenfinches (*Carduelis chloris*) with brighter ornaments have higher virus infection clearance rate. *Behav. Ecol. Sociobiol.*, **48**, 44–51.
López, S. (1998) Acquired resistance affects male sexual display and female choice in guppies. *Proceedings of the Royal Society of London, Series B: Biological Sciences*, **265**, 717–723.
Møller, A.P., P. Christe & E. Lux. (1999) Parasitism, host immune function, and sexual selection. *Q. Rev. Biol.*, **74**, 3–20.
Moran, N.P., A. Sánchez-Tójar, H. Schielzeth & K. Reinhold. (2021) Poor nutritional condition promotes high-risk behaviours: a systematic review and meta-analysis. *Biol. Rev. Camb. Philos. Soc.*, **96**, 269–288.
Nakagawa, S., M. Lagisz, R.E. O'Dea, J. Rutkowska, Y. Yang, D.W.A. Noble, *et al.* (2021) The orchard plot: cultivating a forest plot for use in ecology, evolution, and beyond. *Res. Synth. Methods*, **12**, 4–12.
Nakagawa, S. & E.S.A. Santos. (2012) Methodological issues and advances in biological meta-analysis. *Evol. Ecol.*, **26**, 1253–1274.
Stephenson, J.F., M. Stevens, J. Troscianko & J. Jokela. (2020) The size, symmetry, and color saturation of a male guppy's ornaments forecast his resistance to parasites. *Am. Nat.*, **196**, 597–608.

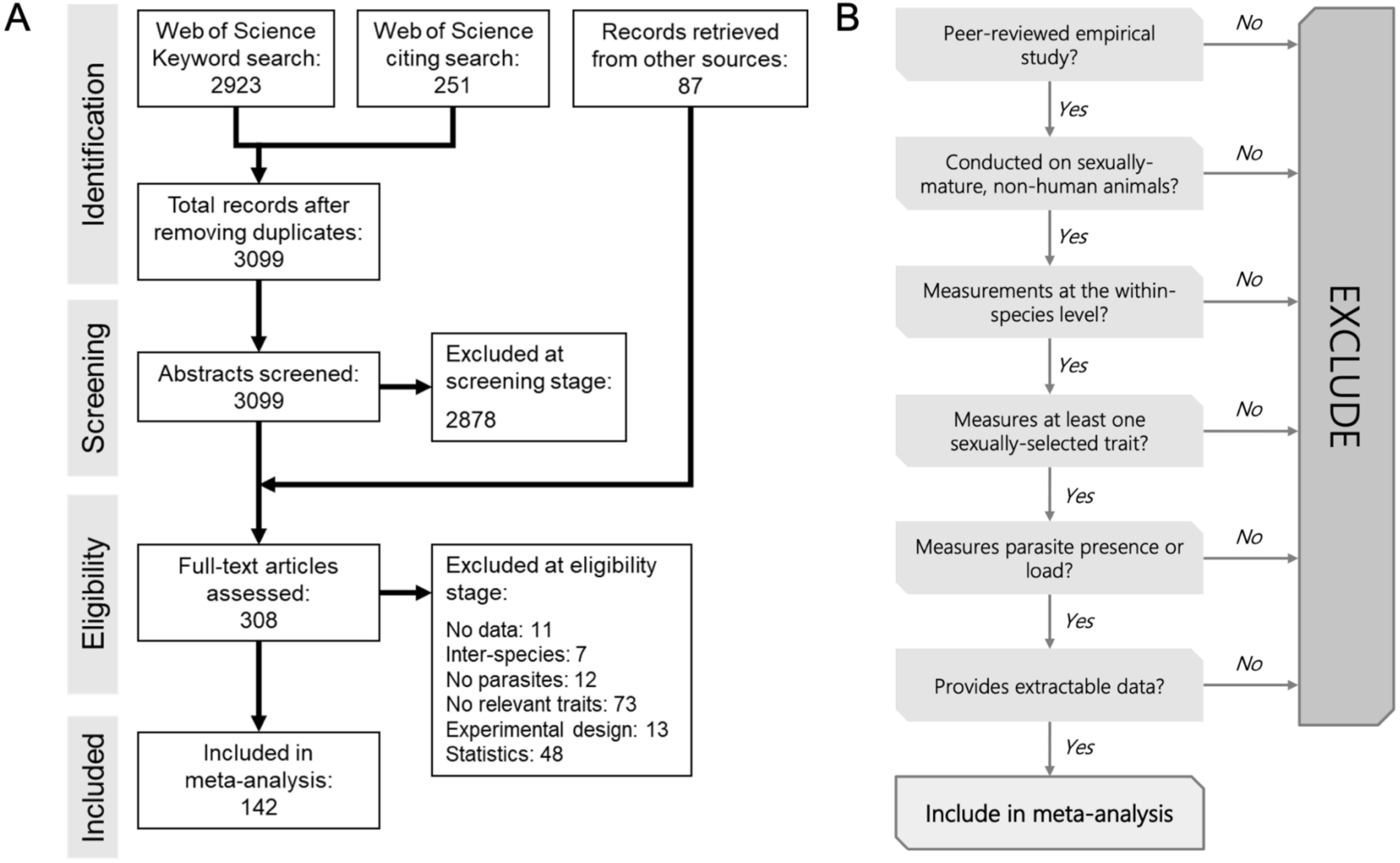

Fig. S1. The literature search and screening process. *PRISMA diagram (a) and diagram showing more detail of the exclusion criteria (b).*

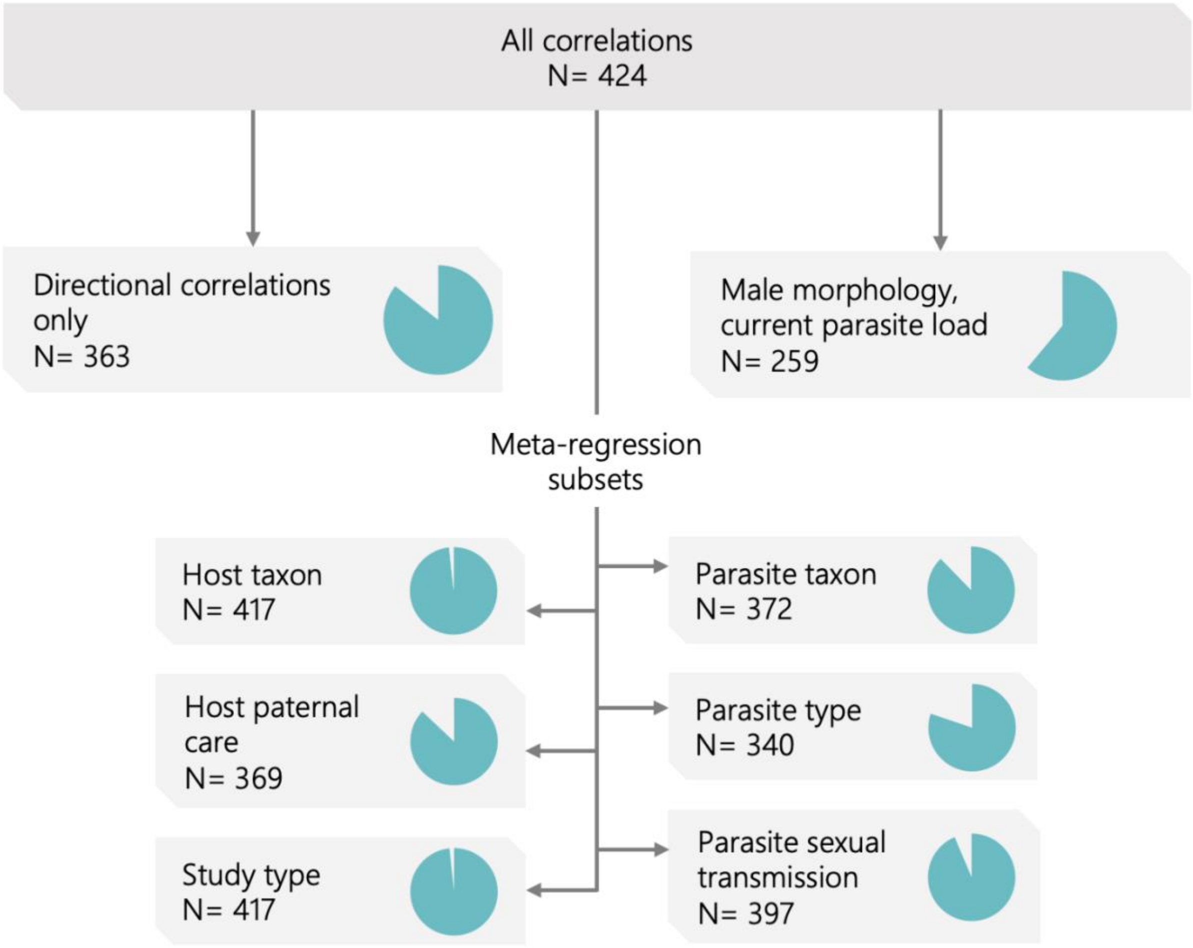

Fig. S2. Subsets of the dataset used during our analyses. *Pie charts represent the proportion of the total sample size that remained in each subset.*

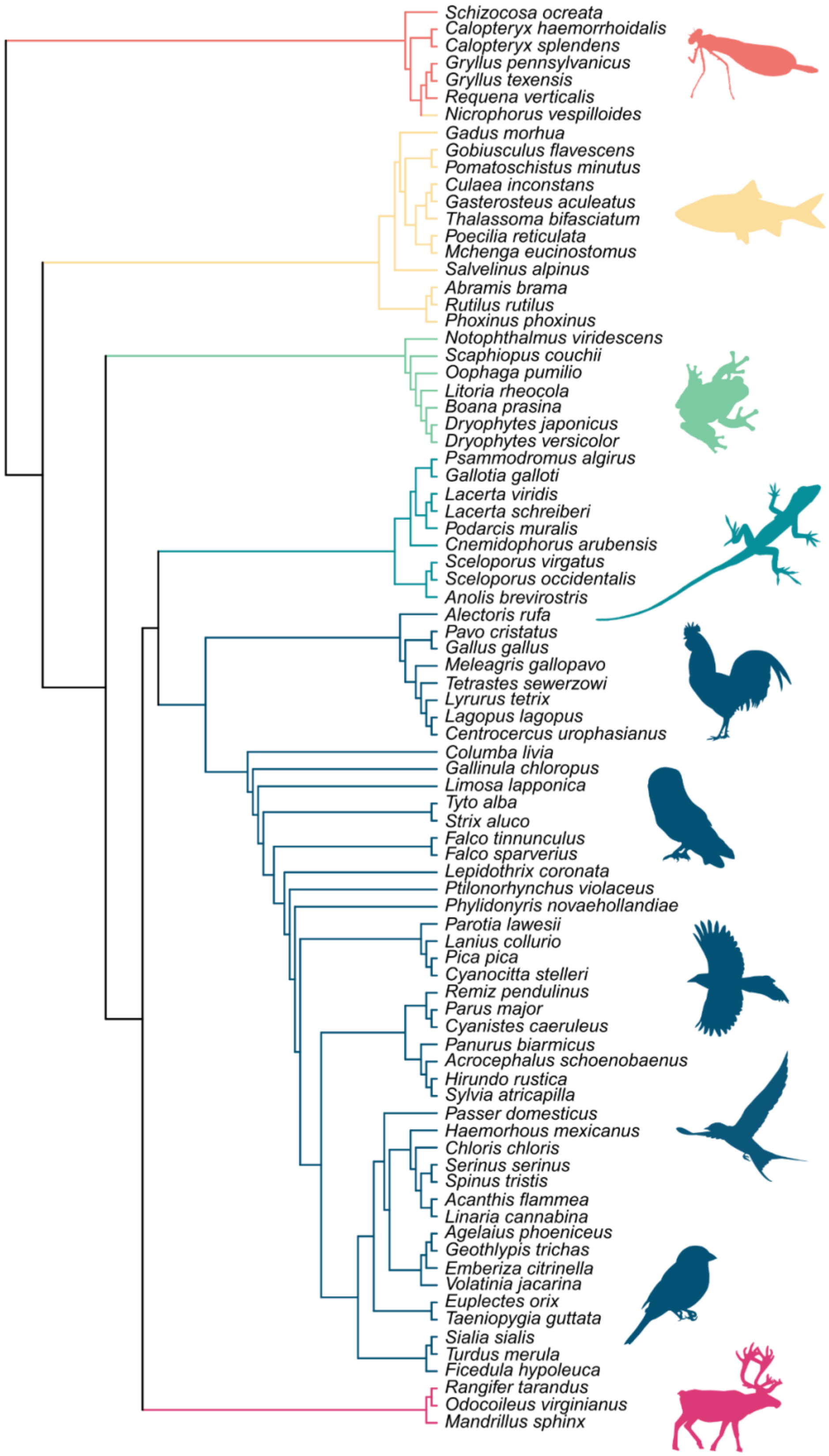

Fig. S3*. *The phylogenetic tree used in the analysis (N = 83 species). *The host taxonomic class categories used during meta-regressions are denoted by the illustrations on the right, and tree branch colours: insects and arachnids (orange), fish (yellow), amphibians (green), reptiles (teal), birds (blue) and mammals (pink).*

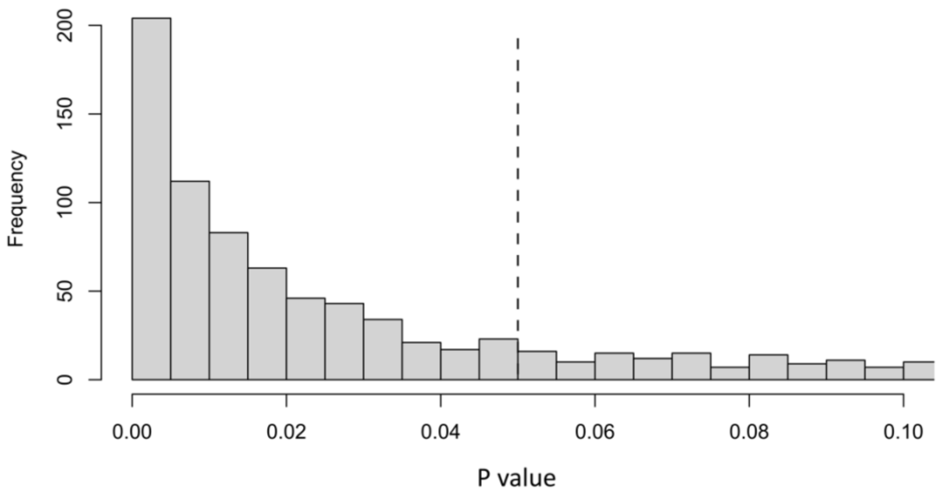

Fig. S4. Histogram showing the distribution of P values from the simulation testing how results from our analysis of only male morphological traits measured at the same time as parasite load (N=259 effect sizes) was affected by a reduction in statistical power compared to our analysis of the full dataset (N=424 effect sizes). *We re-ran the overall meta-analysis model after randomly removing 165 rows from the data set. We did this random removal and re-analysis 1000 times, and find that in 35.8% of cases the P value is greater than 0.05. Across these 1000 datasets, the overall mean correlation between parasite load and ornament quality was -0.083 (bootstrapped 95% confidence intervals: -0.119 to -0.017).*

**Factor**	**Levels**	***k***	**Q_M**	**P**	**Marginal r^2**
Host taxon	6	417	2.95	0.71	0.023
Host sex	2	424	1.28	0.26	0.004
Host paternal care	2	369	0.59	0.44	0.006
Parasite taxon	5	372	2.30	0.68	0.022
Parasite type	2	340	0.64	0.42	0.004
Parasite sexual transmission	3	397	10.87	0.004	0.070
Ornament mate choice	2	424	1.74	0.19	0.009
Ornament dynamism	2	424	5.37	0.02	0.021
Ornament dynamism score	3	424	5.42	0.07	0.021
Parasite measurement	4	424	4.60	0.20	0.021
Study type	2	417	2.37	0.12	0.013
Uninfected	2	424	0.19	0.66	0.001
Precision	NA	424	0.12	0.73	0.000
Study year	NA	424	0.91	0.34	0.005

Table S1. Results from meta-regression models using the full dataset. *Highlighting denotes the two factors that explain significant portions of the heterogeneity.*

Factor	Level	Effect sizes	Studies	Species	Mean *r*	Lower *r*	Upper *r*
Host taxon	Amphibian	20	6	6	0.050	-0.186	0.281
	Arachnid	7	2	1	0.312	-0.131	0.650
	Bird	256	84	45	-0.108	-0.185	-0.029
	Fish	88	25	12	-0.038	-0.186	0.112
	Insect	11	7	6	-0.123	-0.364	0.134
	Mammal	13	5	3	-0.038	-0.343	0.274
	Reptile	29	13	10	-0.175	-0.354	0.017
Host sex	Female	54	22	21	-0.029	-0.140	0.082
	Male	370	132	79	-0.091	-0.153	-0.029
Host paternal care	No	168	58	37	-0.064	-0.160	0.033
	Yes	255	83	45	-0.114	-0.195	-0.031
Parasite taxon	Acanthocephala	2	2	2	0.316	-0.160	0.672
	Arthropod	110	45	30	-0.154	-0.254	-0.050
	Bacteria	32	7	5	0.020	-0.222	0.259
	Cnidaria	10	1	1	-0.122	-0.367	0.138
	Fungi	3	3	3	0.117	-0.281	0.481
	Mollusc	2	1	1	-0.109	-0.591	0.431
	Nematode	59	21	10	-0.071	-0.216	0.077
	Platyhelminth	56	20	14	-0.071	-0.211	0.071
	Protist	115	44	35	-0.123	-0.218	-0.026
	Virus	4	2	2	-0.254	-0.610	0.187
Parasite type	Ectoparasite	157	57	36	-0.127	-0.217	-0.034
	Endoparasite	248	89	61	-0.084	-0.163	-0.004
Parasite risk of sexual transmission	High	142	52	32	-0.141	-0.233	-0.046
	Medium	61	23	19	-0.250	-0.367	-0.126
	No	194	65	43	-0.017	-0.099	0.066
Ornament mate choice	No	241	88	66	-0.062	-0.129	0.007
	Yes	183	63	34	-0.122	-0.203	-0.040
Ornament dynamism	Static	142	51	40	-0.016	-0.098	0.066
	Dynamic	282	104	60	-0.116	-0.183	-0.049
Ornament dynamism score	0	26	12	11	0.009	-0.173	0.174
	1	116	41	32	-0.020	-0.110	0.070
	2	282	96	61	-0.117	-0.185	-0.049
Parasite measurement	Categorical	17	7	6	-0.022	-0.259	0.217
	Group	31	14	12	-0.219	-0.366	-0.062
	Intensity	286	95	60	-0.090	-0.160	-0.020
	Presence / absence	90	32	32	-0.031	-0.132	0.071
Study type	Experimental	53	23	16	-0.177	-0.304	-0.043
	Observational	364	117	77	-0.069	-0.132	-0.006
	Resistance	7	3	2	-0.141	-0.477	0.232
Uninfected hosts present?	No	54	33	25	-0.059	-0.183	0.067
	Yes	370	131	80	-0.088	-0.151	-0.024

Table S2. Mean effect size estimates (r), 95% confidence intervals, and sample sizes for the full data set. *Means for each factor level was obtained using a minus-intercept meta-regression, performed separately for each moderator.*

Factor	Effect sizes	Mean r	Lower r	Upper r	P
All	424	-0.084	-0.143	-0.023	0.007
Males only	370	-0.092	-0.154	-0.029	0.004
Morphological traits only	352	-0.093	-0.159	-0.026	0.006
Simultaneous measurements only	358	-0.070	-0.134	-0.006	0.03
Males, morphological traits, simultaneous measurements only	259	-0.070	-0.143	0.004	0.06

Table S3. Mean effect size estimates (r), 95% confidence intervals, P values, and sample sizes for the different subsets we analysed. *We used re-analyses of our dataset to evaluate the robustness of our results to the breadth of our inclusion criteria. Because removal of female ornamentation, non-morphological traits, and asynchronous measurement of ornaments and parasite load substantially reduced our sample size and thus statistical power, we ran a simulation to test whether this loss of statistical power could explain the loss of the significantly negative overall effect size (see Fig. S4).*

PRISMA-EcoEvo checklist

The PRISMA-EcoEvo extension was published in 2021. It consists of a 27-item checklist and guidance for reporting systematic reviews and meta-analyses of primary research in ecology and evolutionary biology. Within each item, sub-items are given a percentage score (calculated using the Shiny app: https://prisma-ecoevo.shinyapps.io/checklist/). Higher item scores thus indicate that a higher proportion of sub-items are reported in the manuscript.

For more information see the original reference:
O'Dea, R.E., Lagisz, M., Jennions, M.D., Koricheva, J., Noble, D.W., Parker, T.H., Gurevitch, J., Page, M.J., Stewart, G., Moher, D. and Nakagawa, S. (2021), Preferred reporting items for systematic reviews and meta-analyses in ecology and evolutionary biology: a PRISMA extension. Biol Rev. doi:10.1111/brv.12721

Checklist item	Item score	Sub-item number	Sub-item	Reported by authors?	Notes
**Title and abstract**	**100%**	1.1	Identify the review as a systematic review, meta-analysis, or both	Yes	Page 2
		1.2	Summarise the aims and scope of the review	Yes	Page 2
		1.3	Describe the data set	Yes	Page 2
		1.4	State the results of the primary outcome	Yes	Page 2
		1.5	State conclusions	Yes	Page 2
		1.6	State limitations	Yes	Page 2
**Aims and questions**	**100%**	2.1	Provide a rationale for the review	Yes	Page 3
		2.2	Reference any previous reviews or meta-analyses on the topic	Yes	Page 3
		2.3	State the aims and scope of the review (including its generality)	Yes	Page 4, 6
		2.4	State the primary questions the review addresses (e.g. which moderators were tested)	Yes	Page 4, 6
		2.5	Describe whether effect sizes were derived from experimental and/or observational comparisons	Yes	Page 19
**Review registration**	**0%**	3.1	Register review aims, hypotheses (if applicable), and methods in a time-stamped and publicly accessible archive and provide a link to the registration in the methods section of the manuscript. Ideally registration occurs before the search, but it can be done at any stage before data analysis.	No	Review not pre-registered

Checklist item	Item score	Sub-item number	Sub-item	Reported by authors?	Notes
		3.2	Describe deviations from the registered aims and methods	not applicable	Review not pre-registered
		3.3	Justify deviations from the registered aims and methods	not applicable	Review not pre-registered
**Eligibility criteria**	**100%**	4.1	Report the specific criteria used for including or excluding studies when screening titles and/or abstracts, and full texts, according to the aims of the systematic review (e.g. study design, taxa, data availability)	Yes	Pages 17-19
		4.2	Justify criteria, if necessary (i.e. not obvious from aims and scope)	Yes	Pages 17-19
**Finding studies**	**100%**	5.1	Define the type of search (e.g. comprehensive search, representative sample)	Yes	Page 17
		5.2	State what sources of information were sought (e.g. published and unpublished studies, personal communications)	Yes	Page 17
		5.3	Include, for each database searched, the exact search strings used, with keyword combinations and Boolean operators	Yes	Page 17
		5.4	Provide enough information to repeat the equivalent search (if possible), including the timespan covered (start and end dates)	Yes	Page 17
**Study selection**	**100%**	6.1	Describe how studies were selected for inclusion at each stage of the screening process (e.g. use of decision trees, screening software)	Yes	Page 17

Checklist item	Item score	Sub-item number	Sub-item	Reported by authors?	Notes
		6.2	Report the number of people involved and how they contributed (e.g. independent parallel screening)	Yes	Page 17
**Data collection process**	**83%**	7.1	Describe where in the reports data were collected from (e.g. text or figures)	Yes	Pages 19-20
		7.2	Describe how data were collected (e.g. software used to digitize figures, external data sources)	Yes	Pages 19-20
		7.3	Describe moderator variables that were constructed from collected data (e.g. number of generations calculated from years and average generation time)	Yes	Pages 20-23
		7.4	Report how missing or ambiguous information was dealt with during data collection (e.g. authors of original studies were contacted for missing descriptive statistics, and/or effect sizes were calculated from test statistics)	Yes	Pages 19-20
		7.5	Report who collected data	Yes	Page 17
		7.6	State the number of extractions that were checked for accuracy by co-authors	No	
**Data items**	**80%**	8.1	Describe the key data sought from each study	Yes	Page 19
		8.2	Describe items that do not appear in the main results, or which could not be extracted due to insufficient information	No	

Checklist item	Item score	Sub-item number	Sub-item	Reported by authors?	Notes
		8.3	Describe main assumptions or simplifications that were made (e.g. categorising both 'length' and 'mass' as 'morphology')	Yes	Pages 20-23
		8.4	Describe the type of replication unit (e.g. individuals, broods, study sites)	Yes	Pages 17-18
**Assessment of individual study quality**	**100%**	9.1	Describe whether the quality of studies included in the systematic review or meta-analysis was assessed (e.g. blinded data collection, reporting quality, experimental versus observational)	Yes	Pages 22-23
		9.2	Describe how information about study quality was incorporated into analyses (e.g. meta-regression and/or sensitivity analysis)	Yes	Pages 24-25
**Effect size measures**	**100%**	10.1	Describe effect size(s) used	Yes	Page 19
		10.2	Provide a reference to the equation of each calculated effect size (e.g. standardised mean difference, log response ratio) and (if applicable) its sampling variance	Yes	Pages 19-20
		10.3	If no reference exists, derive the equations for each effect size and state the assumed sampling distribution(s)	not applicable	
**Missing data**	**NaN%**	11.1	Describe any steps taken to deal with missing data during analysis (e.g. imputation, complete case, subset analysis)	not applicable	
		11.2	Justify the decisions made to deal with missing data	not applicable	

Checklist item	Item score	Sub-item number	Sub-item	Reported by authors?	Notes
**Meta-analytic model description**	**100%**	12.1	Describe the models used for synthesis of effect sizes	Yes	Pages 23-25
		12.2	The most common approach in ecology and evolution will be a random-effects model, often with a hierarchical/multilevel structure. If other types of models are chosen (e.g. common/fixed effects model, unweighted model), provide justification for this choice	not applicable	
**Software**	**100%**	13.1	Describe the statistical platform used for inference (e.g. R)	Yes	Page 23
		13.2	Describe the packages used to run models	Yes	Page 23
		13.3	Describe the functions used to run models	Yes	Pages 23-24
		13.4	Describe any arguments that differed from the default settings	not applicable	
		13.5	Describe the version numbers of all software used	Yes	Page 23
**Non-independence**	**100%**	14.1	Describe the types of non-independence encountered (e.g. phylogenetic, spatial, multiple measurements over time)	Yes	Pages 23-24
		14.2	Describe how non-independence has been handled	Yes	Pages 23-24
		14.3	Justify decisions made	Yes	Pages 23-24
**Meta-regression and model selection**	**100%**	15.1	Provide a rationale for the inclusion of moderators (covariates) that were evaluated in meta-regression models	Yes	Pages 20-25

Checklist item	Item score	Sub-item number	Sub-item	Reported by authors?	Notes
		15.2	Justify the number of parameters estimated in models, in relation to the number of effect sizes and studies (e.g. interaction terms were not included due to insufficient sample sizes)	not applicable	
		15.3	Describe any process of model selection	not applicable	
**Publication bias and sensitivity analysis**	**100%**	16.1	Describe assessments of the risk of bias due to missing results (e.g. publication, time-lag, and taxonomic biases)	Yes	Page 25
		16.2	Describe any steps taken to investigate the effects of such biases (if present)	Yes	Page 25
		16.3	Describe any other analyses of robustness of the results, e.g. due to effect size choice, weighting or analytical model assumptions, inclusion or exclusion of subsets of the data, or the inclusion of alternative moderator variables in meta-regressions	Yes	Pages 23-24
**Clarification of post hoc analyses**	**100%**	17.1	When hypotheses were formulated after data analysis, this should be acknowledged.	Yes	Page 24
**Metadata, data, and code**		18.1	Share metadata (i.e. data descriptions)	Yes	
		18.2	Share data required to reproduce the results presented in the manuscript	Yes	

Checklist item	Item score	Sub-item number	Sub-item	Reported by authors?	Notes
		18.3	Share additional data, including information that was not presented in the manuscript (e.g. raw data used to calculate effect sizes, descriptions of where data were located in papers)	Yes	
		18.4	Share analysis scripts (or, if a software package with graphical user interface (GUI) was used, then describe full model specification and fully specify choices)	Yes	
**Results of study selection process**	**100%**	19.1	Report the number of studies screened	Yes	Extended data Figure 1
		19.2	Report the number of studies excluded at each stage of screening	Yes	Extended data Figure 1
		19.3	Report brief reasons for exclusion from the full text stage	Yes	Extended data Figure 1
		19.4	Present a Preferred Reporting Items for Systematic Reviews and Meta-Analyses (PRISMA)-like flowchart (www.prisma-statement.org).	Yes	Extended data Figure 1
**Sample sizes and study characteristics**	**60%**	20.1	Report the number of studies and effect sizes for data included in meta-analyses	Yes	Pages 6-11
		20.2	Report the number of studies and effect sizes for subsets of data included in meta-regressions	Yes	Pages 6-11

Checklist item	Item score	Sub-item number	Sub-item	Reported by authors?	Notes
		20.3	Provide a summary of key characteristics for reported outcomes (either in text or figures; e.g. one quarter of effect sizes reported for vertebrates and the rest invertebrates)	Yes	Page 6
		20.4	Provide a summary of limitations of included moderators (e.g. collinearity and overlap between moderators)	No	
		20.5	Provide a summary of characteristics related to individual study quality (risk of bias)	No	
**Meta-analysis**	**100%**	21.1	Provide a quantitative synthesis of results across studies, including estimates for the mean effect size, with confidence/credible intervals	Yes	Pages 8-10
**Heterogeneity**		22.1	Report indicators of heterogeneity in the estimated effect (e.g. I2, tau2 and other variance components)	Yes	Pages 8-9
**Meta-regression**		23.1	Provide estimates of meta-regression slopes (i.e. regression coefficients) and confidence/credible intervals	Yes	Page 11
		23.2	Include estimates and confidence/credible intervals for all moderator variables that were assessed (i.e. complete reporting)	Yes	Extended data FIgure 4
		23.3	Report interactions, if they were included	Yes	Page 10
		23.4	Describe outcomes from model selection, if done (e.g. R2 and AIC)	not applicable	

Checklist item	Item score	Sub-item number	Sub-item	Reported by authors?	Notes
**Outcomes of publication bias and sensitivity analysis**	**100%**	24.1	Provide results for the assessments of the risks of bias (e.g. Egger's regression, funnel plots)	Yes	Page 11
		24.2	Provide results for the robustness of the review's results (e.g. subgroup analyses, meta-regression of study quality, results from alternative methods of analysis, and temporal trends)	Yes	Page 11
**Discussion**	**100%**	25.1	Summarise the main findings in terms of the magnitude of effect	Yes	Page 12
		25.2	Summarise the main findings in terms of the precision of effects (e.g. size of confidence intervals, statistical significance)	Yes	Page 12
		25.3	Summarise the main findings in terms of their heterogeneity	Yes	Pages 14-15
		25.4	Summarise the main findings in terms of their biological/practical relevance	Yes	Pages 12-17
		25.5	Compare results with previous reviews on the topic, if available	Yes	Pages 12-17
		25.6	Consider limitations and their influence on the generality of conclusions, such as gaps in the available evidence (e.g. taxonomic and geographical research biases)	Yes	Pages 12-17
**Contributions and funding**	**100%**	26.1	Provide names, affiliations, and funding sources of all co-authors	Yes	
		26.2	List the contributions of each co-author	Yes	

Checklist item	Item score	Sub-item number	Sub-item	Reported by authors?	Notes
		26.3	Provide contact details for the corresponding author	Yes	
		26.4	Disclose any conflicts of interest	not applicable	
**References**	**100%**	27.1	Provide a reference list of all studies included in the systematic review or meta-analysis	Yes	
		27.2	List included studies as referenced sources (e.g. rather than listing them in a table or supplement)	Yes	
